# Nomograms to predict individual prognosis of patients with squamous cell carcinoma of the urinary bladder

**DOI:** 10.1186/s12885-019-6430-6

**Published:** 2019-12-09

**Authors:** Guanghao Zhang, Zhiwei Li, Daoqing Song, Zhiqing Fang

**Affiliations:** 1 Department of Urology, Qilu Hospital of Shandong University, No. 107 West Wenhua Road, Jinan, Shandong Province China; 2 Department of Biostatistics, School of Public Health, University of Michigan, Ann Arbor, MI USA; 3 Department of Neurosurgery, Qilu Hospital of Shandong University, Jinan, Shandong Province China; 4 Brain Science Research Institute, Shandong University, Jinan, Shandong Province China; 5 Department of Urology, Kunshan People Hospital, Kunshan, Jiangsu Province China

**Keywords:** Squamous cell carcinoma of the bladder, Nomogram, Prognosis, Survival analysis

## Abstract

**Background:**

On the basis of some significant clinical parameters, we had an intent to establish nomograms for estimating the prognosis of patients with squamous cell carcinoma of the urinary bladder (SCCB), including overall survival (OS) and cancer-specific survival (CSS).

**Methods:**

The data of 1210 patients diagnosed with SCCB between 2004 and 2014,were obtained from the Surveillance, Epidemiology, and End Results (SEER) database. The Cox proportional hazards regression model was applied to evaluate the association between variables and survival. Nomograms were constructed to predict the OS and CSS of an individual patient based on the Cox model. In the end, the performance of nomograms was internally validated by using calibration curves, concordance index (C-index), and k-fold cross-validation.

**Results:**

Several common indicators were taken into the two nomograms (OS and CSS), including age at diagnosis, marital status, sex, TNM stage, surgical approach, tumor size, and lymph node ratio while the OS nomogram additionally contained race, grade, and chemotherapy. They had an excellent predictive accuracy on 1- and 3- year OS and CSS with C-index of 0.733 (95% confidence interval [CI], 0.717–0.749) for OS and 0.724 (95% CI, 0.707–0.741) for CSS. All calibration curves showed great consistency between actual survival and predictive survival.

**Conclusions:**

The nomograms with improved accuracy and applicability on predicting the survival outcome of patients with SCCB would provide a reliable tool to help clinicians to evaluate the risk of patients and make individual treatment strategies.

## Background

Urinary bladder carcinoma is one of the most common malignancies with around 77,000 new cases and 16,000 deaths per year in the United States [1]. It has several histological types including transitional cell carcinoma (TCC), squamous cell carcinoma (SCC), adenocarcinoma, small cell carcinoma, and other less common types. Among them, the majority is TCC that accounts for 90–95% of urinary bladder carcinoma, while SCC only accounts for 2–5% [2]. Therefore, researchers naturally have paid more attention to TCC rather than SCC. However, SCC of the bladder has a high degree of malignity and a high incidence of recurrence [3]. Moreover, it is worth noting that for patients with stage III or IV bladder cancer, SCC had a more rapid disease progression than TCC [4]. Hence, it is necessary to understand SCC of the bladder (SCCB) better, especially for prognosis.

Owing to the low incidence of SCCB, an accurate and applicable prognosis model for this disease does not exist. The American Joint Committee on Cancer (AJCC) has established the tumor node metastasis (TNM) staging system, which is the most common method for predicting patients’ prognosis. However, the TNM scheme does not consider factors like demographic information and treatment, which may also prompt a significant association with survival outcomes, although some of them have not thoroughly been studied yet. Spradling et al. reported that lymphovascular invasion was associated with oncologic outcomes for SCCB [5]. A study that involved 45 cases of SCCB [6] shows that radical cystectomy with lymph node dissection appeared to offer a significant benefit to survival in a subset of these SCCB patients. However, Scosyrev et al. reported that SCCB histologic features were not associated with increased mortality among patients with AJCC Stage I and II tumors treated with cystectomy [7]. Abdollah et al. also found a more advanced stage at the surgery for SCCB, but its histological subtype is not associated with a less favorable prognosis than the urothelial carcinoma histological subtype [8]. Besides, several molecular biomarkers have been explored in predicting survival outcomes like fibroblast growth factor 2 (FGF-2), cyclooxygenase 2 (COX-2), p53, Bax, and epidermal growth factor receptor (EGFR) [9–11]. However, the application of molecular biomarkers in prognosis is restricted because of the expenses and inconvenience. In a word, a convenient, comprehensive, and accurate prognostic model is greatly needed by clinicians.

Nomogram is a visible tool based on statistical models that can improve accuracy in predicting prognoses [12]. Many studies have demonstrated that nomograms have higher accuracy than that of risk groups assignment model and staging model because nomograms contain various significant clinical and pathological factors [13–16]. By integrating these factors into nomograms, we can obtain the probability of individual survival outcomes at a specific timepoint. Therefore, nomogram is a reliable tool that could be used to evaluate prognoses and guide decisions on treatment.

Nowadays, there are no valid prognostic models for patients with SCCB, though this is one of the most deadly histological types of urinary bladder carcinoma. This study aims to establish nomograms based on significant clinicopathological parameters (grade, tumor size, TNM stages, LNR), demographic information (age, marriage, sex, race) and therapy (radical cystectomy, chemotherapy) to predict prognostic outcomes of patients with SCCB.

## Methods

### Patient selection

In this study, all data of patients were obtained from the Surveillance, Epidemiology, and End Results (SEER) database, which collects and publishes data including cancer incidence and mortality from 20 cancer registries that cover approximately 28% of the population of the United States. The study cohort consists of patients who met the following criteria: 1) age at diagnosis between 18 and 100 years old; 2) positive histology; 3) histological type limited to squamous cell carcinoma of the bladder (ICD-O-3 codes: 8070/3, 8072/3, 8074/3–8077/3); 4) active follow-up with complete date and known survival months and known cause of death; 5) adequate/consistent information on variables including age at diagnosis, sex, race, marital status, Fuhrman grade, TNM stage, number of regional lymph node removed, number of regional lymph node positive, surgery of the primary tumor, surgery of metastasis, radiation, and chemotherapy. Patients in the cohort diagnosed before 2004 were excluded since their TNM stage information was not recorded in the SEER database. After selection, 1210 eligible patients were enrolled in the cohort.

### Variables

The variables analyzed in this study were age at diagnosis, sex, race, marital status, Fuhrman grade, pathological stage (T/N/M, derived AJCC, sixth edition), surgery of the primary tumor, radiation, chemotherapy, and metastasectomy. Some of the variables were regrouped in the analysis. Patients with specific age at diagnosis were regrouped into “< 50”, “50–59”, “60–69”, “70–79”, “80–89”, and “90–100”. Patients whose race was recorded as American Indian/Alaskan Native or Asian/ Pacific Islander were assigned to an “others” race category. Patients whose marital status was recorded as “Divorced”, “Single” or “Widowed” in the SEER database were regrouped into “Single”. The surgical treatment variable was grouped into “Yes” (Radical cystectomy: RX Summ-Surg Prim Site code < 30) and “No” (non-radical cystectomy: RX Summ-Surg Prim Site code 50–80). The T stage was regrouped into Ta, Tis, T1, T2 (T2a/T2b/T2NOS), T3 (T3a/T3b/T3NOS), and T4 (T4a/T4b/T4NOS). Additionally, considering that the lymph node ratio (LNR) has been commonly used as a quality indicator in bladder cancer, LNR was calculated by dividing the positive node number by the examined node number [17–19]. To evaluate the prognostic value of lymph node ratio in SCCB patients, positive LNR was stratified into two categories (cut-off point 0.0385) by X-tile program, which is a practical tool for cut-point optimization [20]. Hence, the variable LNR was divided into four categories: LNR = 0 (patients did not receive lymphadenectomy), 0 < LNR ≤ 0.0385, LNR > 0.0385, and unknown. Using a similar approach, we identified 6.5 cm as the cut-off point for the size of the tumor of patients in the cohort. The primary endpoints of the study were overall survival (OS) and cancer-specific survival (CSS). Survival time was calculated from the date of diagnosis to the date of 1) death from any cause for OS; 2) death from SCCB for CSS; or 3) the last follow-up. Frequency and proportion were reported for each variable analyzed in this study.

### Statistical analysis

The univariable Cox regression analyses were firstly used to verify whether the association between each variable and survival outcomes, including OS and CSS, is significant. After removing insignificant variables, the multivariable Cox regression analyses were then employed to calculate the association between variables and survival outcomes, including OS and CSS. The variables incorporated into the multivariable Cox models were checked whether they fit the proportional hazards (PH) assumption and found that several variables, including surgery type, T, and N, violated the PH assumption in both OS and CSS models. However, these variables are significant according to the univariable Cox analyses, and in consideration of their clinical significance and their presences improving the fit of the model, we included them in the multivariable Cox analyses. The measure of the association was presented as a hazard ratio (HR). Nomograms in this study were created using information obtained from the multivariable Cox regression analyses.

To decrease the overfit bias, internal validation of this nomogram was then performed using the .632+ bootstrap method with 150 resamples. Predictive performance was then assessed by using the concordance index (C-index). Calibration curves of the nomograms were derived to evaluate the consistency between predicted survival and observed survival. In addition, the 6-fold cross-validation method was applied to evaluate the performance of our multivariable Cox regression models internally.

In the R software (Version 3.5.3), the Cox regression analyses were performed by using the survival package and rms package, the nomogram was graphed by using the rms package, validation was performed by using the rms package, and the 6-fold cross-validation was performed by using the hdnom package. All statistical tests were considered statistically significant at *P* < 0.05.

## Results

### Patients baseline characteristics

According to the inclusion criteria, we selected a total of 1210 patients in this study. Demographics, tumor, and therapy characteristics of the cohort are listed in Table 1. Generally, the majority of patients were Caucasian (1009, 83.39%) and older than 50 years old (1104, 91.24%) with grade III (480, 39.67%). With regard to therapy, most patients did not have radical cystectomy (728, 60.17%), metastasectomy (1128, 93.22%), radiation (993, 82.07%), or chemotherapy (891, 73.64%). The initial analysis results showed that the 1-year and 3-year OS rates were 40.91 and 29.10%, respectively, while the rates for CSS were 46.03 and 34.30%, respectively.

**Table 1:** Demographics, tumor and therapy characteristics of the selected cohort

Variables	No.	%
Cases evaluated	1210	100
Age at diagnosis		
< 50	106	8.76
50–59	194	16.03
60–69	253	20.91
70–79	326	26.94
80–89	278	22.98
90–100	53	4.38
Marital status		
Married	532	43.97
Single	630	52.07
Unknown	48	3.97
Race		
Black	149	12.31
White	1009	83.39
Other	52	4.30
Sex		
Female	619	51.16
Male	591	48.84
Grade		
I	107	8.84
II	462	38.18
III	480	39.67
IV	161	13.31
T		
T1	163	13.47
T2	474	39.17
T3	292	24.13
T4	266	21.98
Ta	6	0.50
Tis	9	0.74
N		
N0	1008	83.31
N1	103	8.51
N2	96	7.93
N3	3	0.25
M		
M0	1091	90.17
M1	119	9.83
Radical Cystectomy		
No	728	60.17
Yes	482	39.83
Metastasectomy		
No	1128	93.22
Yes	82	6.78
Radiation		
No/unknown	993	82.07
Yes	217	17.93
Chemotherapy		
No/unknown	891	73.64
Yes	319	26.36
Size		
≤ 6.5 cm	470	38.84
>6.5 cm	303	25.04
Unknown	437	36.12
LNR		
0	339	28.02
≤ 0.0385	11	0.91
>0.0385	111	9.17
Unknown	749	61.90

### Univariable and multivariable cox regression in the cohort

We used univariable and multivariable Cox regression to analyze the association between these selected characteristics with OS or CSS. As shown in Table 2, in univariable Cox regression analysis for OS, characteristics reaching statistical significance were as follows: age at diagnosis, marital status, race, sex, grade, TNM stage, radical cystectomy, chemotherapy, tumor size, and LNR. However, in multivariable Cox regression for CSS, race, grade, and chemotherapy were statistically insignificant. Then, we incorporated variables that were significant in the univariable Cox regressions into the multivariable Cox regressions for OS and CSS, respectively.

**Table 2:** Univariate Cox regression model analysis for overall survival and cancer specific survival in nomogram cohort

Characteristics	Overall Survival (OS)		Cancer Specific Survival (CSS)	
	HR^a^ (95% CI^b^)	*P*-value	HR (95% CI)	*P*-value
Age at diagnosis				
< 50	1 (Reference)		1 (Reference)	
50–59	0.842 (0.632–1.121)	0.239	0.782 (0.581–1.053)	0.105
60–69	0.861 (0.654–1.134)	0.288	0.746 (0.560–0.995)	0.046
70–79	1.040 (0.803–1.349)	0.765	0.876 (0.668–1.149)	0.338
80–89	1.655 (1.276–2.146)	< 0.001	1.313 (1.000–1.725)	0.050
90–100	2.447 (1.701–3.520)	< 0.001	1.983 (1.341–2.931)	0.001
Marital status				
Married	1 (Reference)		1 (Reference)	
Single	1.756 (1.528–2.017)	< 0.001	1.761 (1.511–2.052)	< 0.001
Unknown	1.614 (1.163–2.240)	0.004	1.610 (1.122–2.312)	0.010
Race				
Black	1 (Reference)		1 (Reference)	
White	0.827 (0.678–1.008)	0.060	0.809 (0.651–1.005)	0.055
Other	0.624 (0.423–0.920)	0.017	0.696 (0.464–1.044)	0.079
Sex				
Female	1 (Reference)		1 (Reference)	
Male	0.773 (0.677–0.883)	< 0.001	0.746 (0.644–0.864)	< 0.001
Grade				
I	1 (Reference)		1 (Reference)	
II	0.943 (0.772–1.151)	0.564	0.903 (0.727–1.121)	0.354
III	0.825 (0.700–0.972)	0.021	0.855 (0.715–1.023)	0.086
IV	0.867 (0.769–0.976)	0.019	0.905 (0.794–1.032)	0.135
T				
T1	1 (Reference)		1(Reference)	
T2	0.939 (0.760–1.161)	0.562	0.947 (0.746–1.201)	0.652
T3	0.634 (0.501–0.802)	< 0.001	0.656 (0.504–0.853)	0.002
T4	1.473 (1.175–1.846)	0.001	1.598 (1.246–2.050)	< 0.001
Ta	0.492 (0.181–1.334)	0.163	0.486 (0.154–1.537)	0.220
Tis	0.928 (0.453–1.902)	0.839	0.576 (0.212–1.570)	0.281
N				
N0	1 (Reference)		1 (Reference)	
N1	1.654 (1.332–2.054)	< 0.001	1.810 (1.439–2.277)	< 0.001
N2	1.707 (1.352–2.154)	< 0.001	1.933 (1.514–2.466)	< 0.001
N3	2.608 (0.837–8.122)	0.098	3.189 (1.023–9.941)	0.046
M				
M0	1 (Reference)		1 (Reference)	
M1	3.071 (2.509–3.758)	< 0.001	3.439 (2.786–4.246)	< 0.001
RC^**c**^				
No	1 (Reference)		1 (Reference)	
Yes	0.400 (0.347–0.462)	< 0.001	0.389 (0.332–0.456)	< 0.001
Metastasectomy				
No	1 (Reference)		1 (Reference)	
Yes	1.009 (0.775–1.315)	0.944	0.991 (0.739–1.328)	0.950
Radiation				
No/unknown	1 (Reference)		1 (Reference)	
Yes	1.116 (0.946–1.316)	0.192	1.132 (0.946–1.355)	0.175
Chemotherapy				
No/unknown	1 (Reference)		1 (Reference)	
Yes	0.824 (0.709–0.956)	0.011	0.870 (0.740–1.023)	0.092
Size				
≤ 6.5 cm	1 (Reference)		1 (Reference)	
>6.5 cm	1.736 (1.459–2.065)	< 0.001	1.806 (1.492–2.187)	< 0.001
Unknown	2.119 (1.815–2.474)	< 0.001	2.151 (1.812–2.555)	< 0.001
LNR^**d**^				
0	1 (Reference)		1(Reference)	
≤ 0.0385	1.796 (0.842–3.830)	0.130	1.730 (0.706–4.240)	0.230
>0.0385	3.307 (2.562–4.269)	< 0.001	4.164 (3.145–5.514)	< 0.001
Unknown	3.293(2.761–3.927)	< 0.001	3.805 (3.098–4.674)	< 0.001

^a^*HR* Hazard ratio

^b^*CI* Confidence interval

^c^*RC* Radical cystectomy

^d^*LNR* Lymph node ratio

As shown in Table 3, multivariable Cox regression analysis for OS indicated that all selected variables had statistical significance except N stage and race. In the multivariable Cox regression analysis for CSS, significant variables were fewer than that in OS, including age at diagnosis, marital status, sex, T stage, M stage, radical cystectomy, tumor size, and LNR. According to Tables 2 and 3, prognostic outcomes and mortality risk of patients can be intuitively evaluated. For example, older patients may have higher possibilities to experience worse OS and CSS outcomes. Similarly, single women are more likely to have poor prognoses. As for therapy, a radical cystectomy may help patients to get a favorable outcome for both OS and CSS, as other studies reported [3].

**Table 3:** Multivariate Cox regression model analyses of overall survival and cancer specific survival in the nomogram cohort

Characteristics	Overall Survival (OS)		Cancer Specific Survival (CSS)	
	HR^a^ (95% CI^b^)	*P*-value	HR (95% CI)	*P*-value
Age at diagnosis				
< 50	1(Reference)		1(Reference)	
50–59	1.118 (0.833–1.499)	0.457	0.981 (0.725–1.328)	0.902
60–69	1.321 (0.990–1.764)	0.059	1.161 (0.856–1.573)	0.338
70–79	1.434 (1.089–1.888)	0.010	1.310 (0.981–1.750)	0.067
80–89	1.935 (1.458–2.568)	< 0.001	1.703 (1.263–2.297)	< 0.001
90–100	2.181 (1.473–3.228)	< 0.001	2.115 (1.388–3.222)	< 0.001
Marital status				
Married	1 (Reference)		1 (Reference)	
Single	1.364 (1.176–1.583)	< 0.001	1.364 (1.16–1.604)	< 0.001
Unknown	1.338 (0.956–1.872)	0.090	1.325 (0.916–1.915)	0.135
Race				
Black	1 (Reference)		Not selected	
White	1.007 (0.819–1.238)	0.946		
Other	0.811 (0.545–1.207)	0.302		
Sex				
Female	1 (Reference)		1 (Reference)	
Male	0.828 (0.719–0.953)	0.009	0.790 (0.677–0.922)	0.003
Grade				
I	1 (Reference)		Not selected	
II	1.112 (0.904–1.368)	0.314		
III	0.772 (0.652–0.914)	0.003		
IV	0.995 (0.879–1.125)	0.932		
T				
T1	1 (Reference)		1(Reference)	
T2	1.394 (1.117–1.741)	0.003	1.304 (1.020–1.665)	0.034
T3	1.660 (1.265–2.177)	< 0.001	1.685 (1.251–2.270)	0.001
T4	2.234 (1.726–2.892)	< 0.001	2.104 (1.589–2.786)	< 0.001
Ta	0.475 (0.174–1.299)	0.147	0.500 (0.157–1.593)	0.241
Tis	0.815 (0.392–1.695)	0.584	0.560 (0.204–1.541)	0.262
N				
N0	1 (Reference)		1 (Reference)	
N1	1.354 (0.987–1.858)	0.060	1.241 (0.892–1.725)	0.199
N2	1.382 (1.013–1.886)	0.041	1.307 (0.951–1.795)	0.098
N3	1.439 (0.443–4.675)	0.545	1.907 (0.587–6.197)	0.283
M				
M0	1 (Reference)		1 (Reference)	
M1	2.358 (1.881–2.956)	< 0.001	2.285 (1.816–2.876)	< 0.001
RC^c^				
No	1 (Reference)		1 (Reference)	
Yes	0.433 (0.342–0.548)	< 0.001	0.436 (0.333–0.572)	< 0.001
Chemotherapy				
No/unknown	1 (Reference)		Not selected	
Yes	0.605 (0.514–0.712)	< 0.001		
Size				
≤ 6.5 cm	1 (Reference)		1 (Reference)	
>6.5 cm	1.815 (1.510–2.182)	< 0.001	1.840 (1.506–2.248)	< 0.001
Unknown	1.509 (1.277–1.783)	< 0.001	1.510 (1.255–1.816)	< 0.001
LNR^d^				
0	1 (Reference)		1 (Reference)	
≤ 0.0385	1.316 (0.578–2.993)	0.513	1.195 (0.460–3.100)	0.714
>0.0385	2.341 (1.629–3.363)	< 0.001	2.797 (1.895–4.130)	< 0.001
Unknown	1.734 (1.328–2.263)	< 0.001	1.846 (1.359–2.509)	< 0.001

^a^*HR* Hazard ratio

^b^*CI* Confidence interval

^c^*RC* Radical cystectomy

^d^*LNR* Lymph node ratio

### Prognostic nomograms for OS and CSS and validations

All of the variables in multivariable Cox regression analyses were taken into consideration in nomograms for 1- and 3- year OS and CSS, which were shown in Additional file 1: Figure S1 and Additional file 2: Figure S2, respectively. Each of the variables was given a point according to HR. Then, by adding up the total score from each variable and locating it onto the total points scale, the probability of 1- and 3- year OS and CSS will be obtained. With the nomogram for OS, one can conclude that if a 65-year old married white man with gradeII, T3N1M0, LNR equals 0, and 1.0 cm size of tumor has taken radical cystectomy and chemotherapy, he would score 151 points, which means that this patient has approximately 80% possibility of survival in the first year and approximately 70% possibility of survival in the third year.

**Fig. 1:**
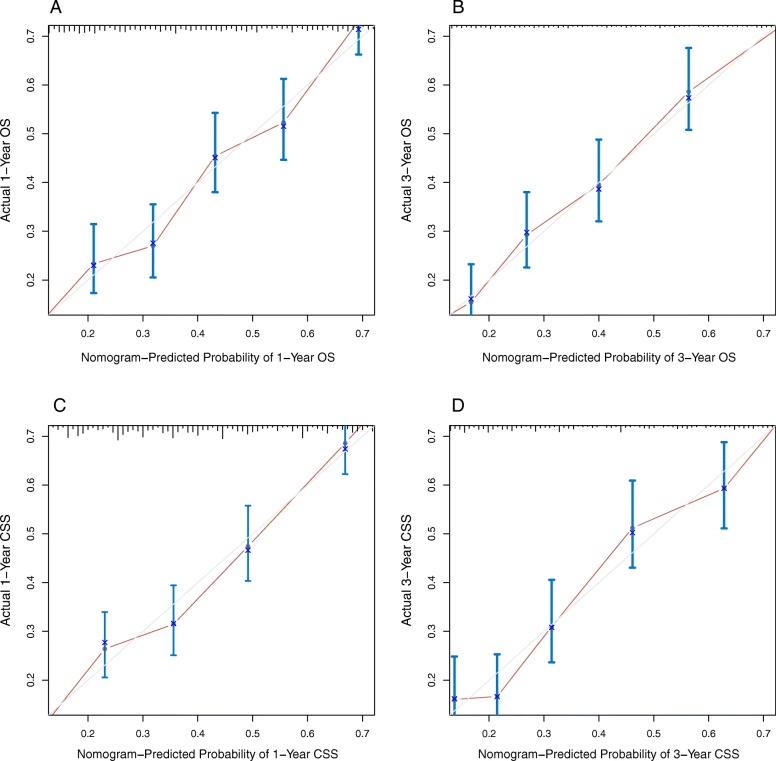
Nomogram model calibration curves: **a**, **b** The internal calibration curves of 1- and 3- year OS as well as (**c**, **d**) The internal calibration curves of 1- and 3- year CSS. Nomogram-predicted probability of survival was plotted on the X-axis, and the actual probability of survival was plotted on the Y-axis. The perfect calibration model was represented by dashed lines which indicated actual probability was exactly the same as predicted probability. The distance between solid lines and dashed lines represented the fitness of actual and nomogram-predicted prognosis. Abbreviations: OS, overall survival; CSS, cancer specific survival

Validation of the nomograms was processed in the internal cohort. The C-indices of the nomograms were 0.733 (95% CI, 0.717–0.749) and 0.724 (95% CI, 0.707–0.741) for OS and CSS respectively, which were both higher than 0.7, suggesting that these two nomograms were relatively accurate and suitable for predicting OS and CSS for patients with SCCB. Moreover, 6-fold cross-validation also showed consistent results in Additional file 3: Figure S3. Internal calibration plots for 1- and 3-year OS and CSS were shown in Fig. 1**,** which revealed the significant correlation between predicted survival and actual survival.

## Discussion

Bladder cancer has approximately 430,000 new diagnoses in the world annually. However, researchers do not have a clear understanding of the prognosis of the SCC subtype because that SCC only accounts for 2–5% of the total cases [2, 7, 21]. In fact, SCCB is divided into two types: bilharzial-associated SCC (B-SCC) and non-bilharzial-associated SCC (NB-SCC) [3]. In the USA, many studies have shown that the majority of SCCB is NB-SCC, which has a worse prognosis than TCC when adjusting for pathological characteristics like TNM staging [22–24]. Jason et al. analyzed 178 cases of pure SCC and 2884 cases of pure UC, finding that SCC led to a more rapid disease progression than that of UC, but they nearly had the same survival outcomes [4]. In regard to treatment, due to lack of high-quality observational studies, there has been no agreement on therapy strategies for SCCB except radical cystectomy and urinary diversion up till now [25].Based on the above reasons, an accurate prognostic prediction model for SCCB patients might have a great clinical value.

However, as a result of the rarity of SCCB patients, there has been no widely accepted predicting model so far. To a certain extent, the AJCC staging system has abilities to predict prognosis, mainly based on T, N, and M information. Nevertheless, it is not specially designed for SCCB, and many individualized characteristics which may be predictive are not involved [26–28]. By contrast, prognostic nomogram is a visualized statistical tool with several advantages, including accuracy, comprehensibility, convenience, and user-friendliness. Hence, nomogram is one of the most widely applied and accurate predictive tools in clinical practice [29, 30]. Currently, there are some nomograms for bladder cancer indeed, but no one is developed for SCCB specifically. Therefore, the two prognostic nomograms for SCCB patients established in this study should be quite useful and practical for clinicians.

Our nomograms are innovative and rational in the following aspects. Firstly, our nomograms are the first method to predict the prognosis of SCCB patients, which makes the individualized prediction of OS and CSS and individualized treatment guidance possible for patients with SCCB in clinical practice. Secondly, many characteristics are involved in our analysis, not only the TNM stage but also other variables like demographic characteristics, clinicopathological parameters, and therapy strategies. According to previous studies, some characteristics did influence the prognosis of bladder cancer. For example, Zahoor et al. revealed that older age (≥70 years old) was associated with worse survival outcomes [31]. Similarly, older groups were assigned with higher points in our nomograms. As for clinicopathological parameters, many studies have discussed the influence of lymph nodes metastasis, staging, grades, and tumor size. Balci et al. found that lymph node involvement, TNM staging, and grade were all critical prognostic factors [32]. Li et al. also showed that tumor size and lymphovascular invasion were the keys to survival outcomes [33]. Additionally, it is noteworthy that radical cystectomy is the ‘gold standard’ of treatment strategies, which provides patients with a better prognosis than partial cystectomy [3]. Moreover, receiving radiation is associated with poor survival in most of the reported studies [34, 35], while radiation does provide some disease-free survival benefit for patients with SCCB based on several studies [36–38]. Chemotherapy is also a fundamental therapy for bladder cancer. Compared to the sensitivity of TCC to chemotherapy, SCC is more resistant to this therapy [39]. However, a study in the U.S. showed that non-TCC could also get benefit from chemotherapy [40]. Our Cox regression analysis for OS also showed that SCCB patients without chemotherapy would experience higher death risk (HR = 1.652, *P* < 0.001). In other words, previous studies have shown that involving these variables in our prognostic model would help to improve accuracy. Thirdly, as a result of the relevant large scale of data from the SEER program and rigorous algorithm, the performance of nomograms was reliable with C-indices of 0.733 (95% CI, 0.717–0.749) and 0.724 (95% CI, 0.707–0.741) for OS and CSS respectively. Hence, these two nomograms are both relatively accurate. Finally, as we have described above, except for the common variables, we have also included some characteristics to analyze their associations with prognosis based on our clinical experience, including marital status, race, sex, metastasectomy, and LNR. It was the first time to show that these variables could be prognostic factors for SCCB patients. Above all, our prognosis models are innovative and rational enough to be useful in clinical practice.

However, there are still several limitations. First of all, our analysis was based on the SEER database, and some accurate information is missing. For example, two categories (“No/Unknown” or “Yes”) were assigned to chemotherapy, which may lead to information bias and influence HR of variables. Additionally, an individual’s social-economic status are not included in SEER database [41], such as education and family income levels, which may also be associated with the prognosis of bladder cancer patients [42]. Therefore, a more dedicated model that includes the social-economic status is in need in the future. Secondly, due to the nature of the retrospective study, these nomograms need to be validated in a prospective cohort in the next step before being formally applied in clinical practice. Finally, despite the C-indices of the two nomograms are greater than 0.7, suggesting high accuracy on OS and CSS, it is not perfect. We still have around 30% of predictions that will be made incorrectly. Indeed, it is impossible to achieve 100% accuracy for any predicting model, but we would try our best to improve the quality and quantity of data and the reliability of algorithms to achieve this aim.

## Conclusions

In this study, our prognostic model revealed that several demographic characteristics, clinicopathological parameters, and therapy strategies have significant associations with survival outcomes of SCCB patients. More importantly, we established the accurate and visible nomograms to predict individual OS and CSS of SCCB patients. The nomograms will help clinicians to evaluate the risk of SCCB patients and apply the individualized treatment.

## Supplementary information

**Additional file 1 **: **Figure S1.** Nomogram for predicting 1- and 3-year OS of SCCB. Instruction of the nomogram: firstly, make a vertical line from certain variable to points scale to assign the point of that characteristic; then, add up all of the points from each characteristic and locating it to the total points’ scale; finally, draw a vertical line from the total points to 1- and 3-year OS to predict the probability of OS at 1- and 3-year. Abbreviations: OS, overall survival; MS, marital status; LNR, lymph node ratio; Sur. Prob., survival probability; SCCB, squamous cell carcinoma of the urinary bladder.

**Additional file 2 **: **Figure S2**. Nomogram for predicting 1- and 3-year CSS of SCCB. Instruction of the nomogram: firstly, make a vertical line from certain variable to points scale to assign the point of that characteristic; then, add up all of the points from each characteristic and locating it to the total points’ scale; finally, draw a vertical line from the total points to 1- and 3-year CSS to predict the probability of CSS at 1- and 3-year. Abbreviations: CSS, cancer specific survival; MS, marital status; LNR, lymph node ratio; Sur. Prob., survival probability; SCCB, squamous cell carcinoma of the urinary bladder.

**Additional file 3 **: **Figure S3.** Time-dependent AUC values for internal model validation. The 6-fold cross validation of (A) OS and (B) CSS. Abbreviations: AUC, area under the curve; OS, overall survival; CSS, cancer specific survival.

## Data Availability

Data for this study were obtained from the US NCI SEER database (https:// seer.cancer.gov).

## Abbreviations

AJCC: American Joint Committee on Cancer
B-SCC: Bilharzial-associated SCC
CI: Confidence interval
C-index: Concordance index
CSS: Cancer specific survival
HR: Hazard ratio
LNR: Lymph node ratio
MS: Marital status
NB-SCC: Non-bilharzial-associated SCC
OS: Overall survival
RC: Radical cystectomy
SCC: Squamous cell carcinoma
SCCB: Squamous cell carcinoma of the bladder
SEER: Surveillance, Epidemiology, and End Results
Sur. Prob.: Survival probability
TCC: Transitional cell carcinoma
UC: Urothelial carcinoma
