# Supplementary figures and images for: Nomograms to predict individual prognosis of patients with squamous cell carcinoma of the urinary bladder
Source: BMC Cancer. 2019 Dec 9;19:1200. doi: 10.1186/s12885-019-6430-6 (PMC6902456; doi:10.1186/s12885-019-6430-6)

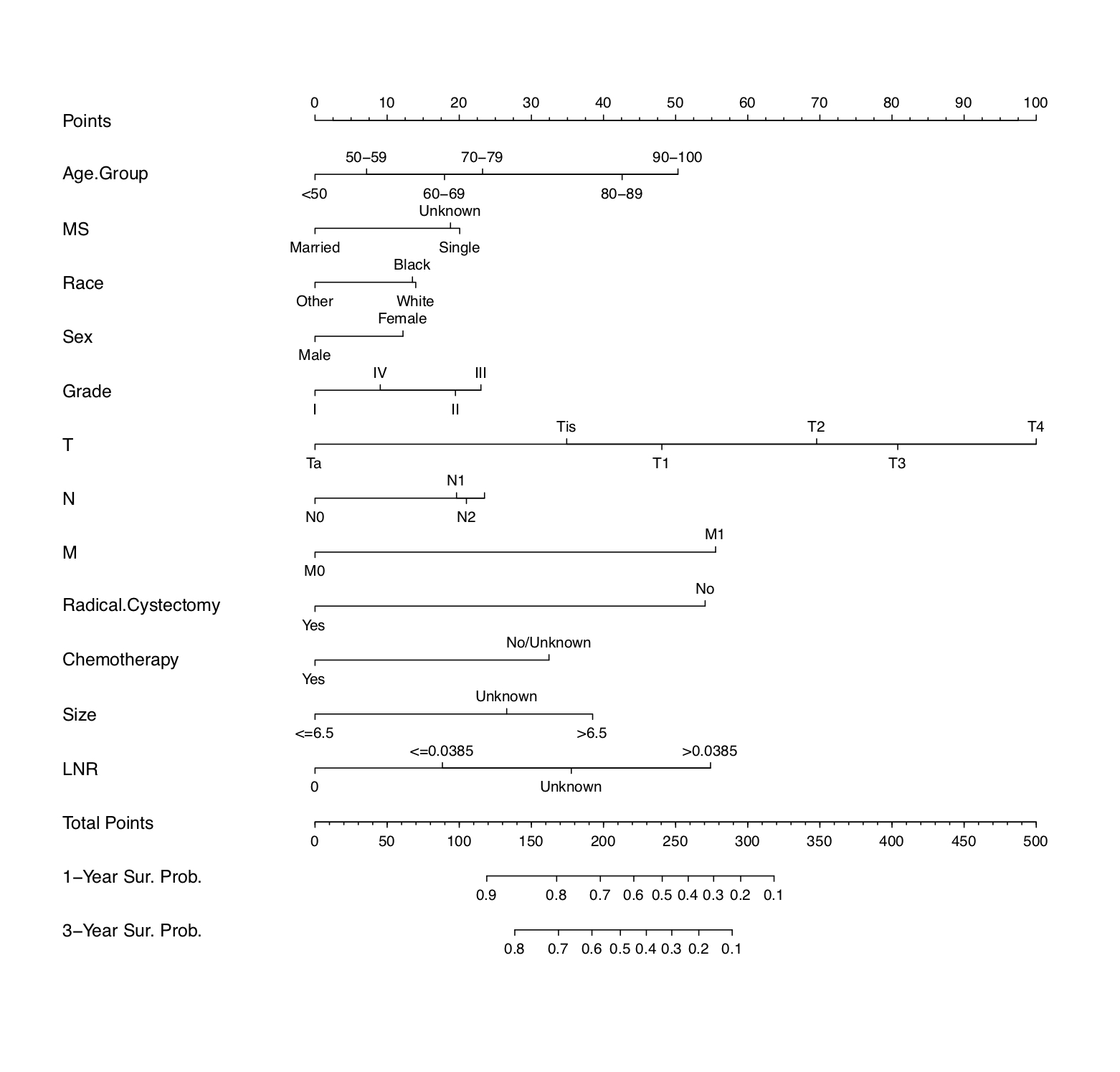

Supplement: Supplementary file 1 — Additional file 1 : Figure S1. Nomogram for predicting 1- and 3-year OS of SCCB. Instruction of the nomogram: firstly, make a vertical line from certain variable to points scale to assign the point of that characteristic; then, add up all of the points from each characteristic and locating it to the total points’ scale; finally, draw a vertical line from the total points to 1- and 3-year OS to predict the probability of OS at 1- and 3-year. Abbreviations: OS, overall survival; MS, marital status; LNR, lymph node ratio; Sur. Prob., survival probability; SCCB, squamous cell carcinoma of the urinary bladder. [file 12885_2019_6430_MOESM1_ESM.jpg]

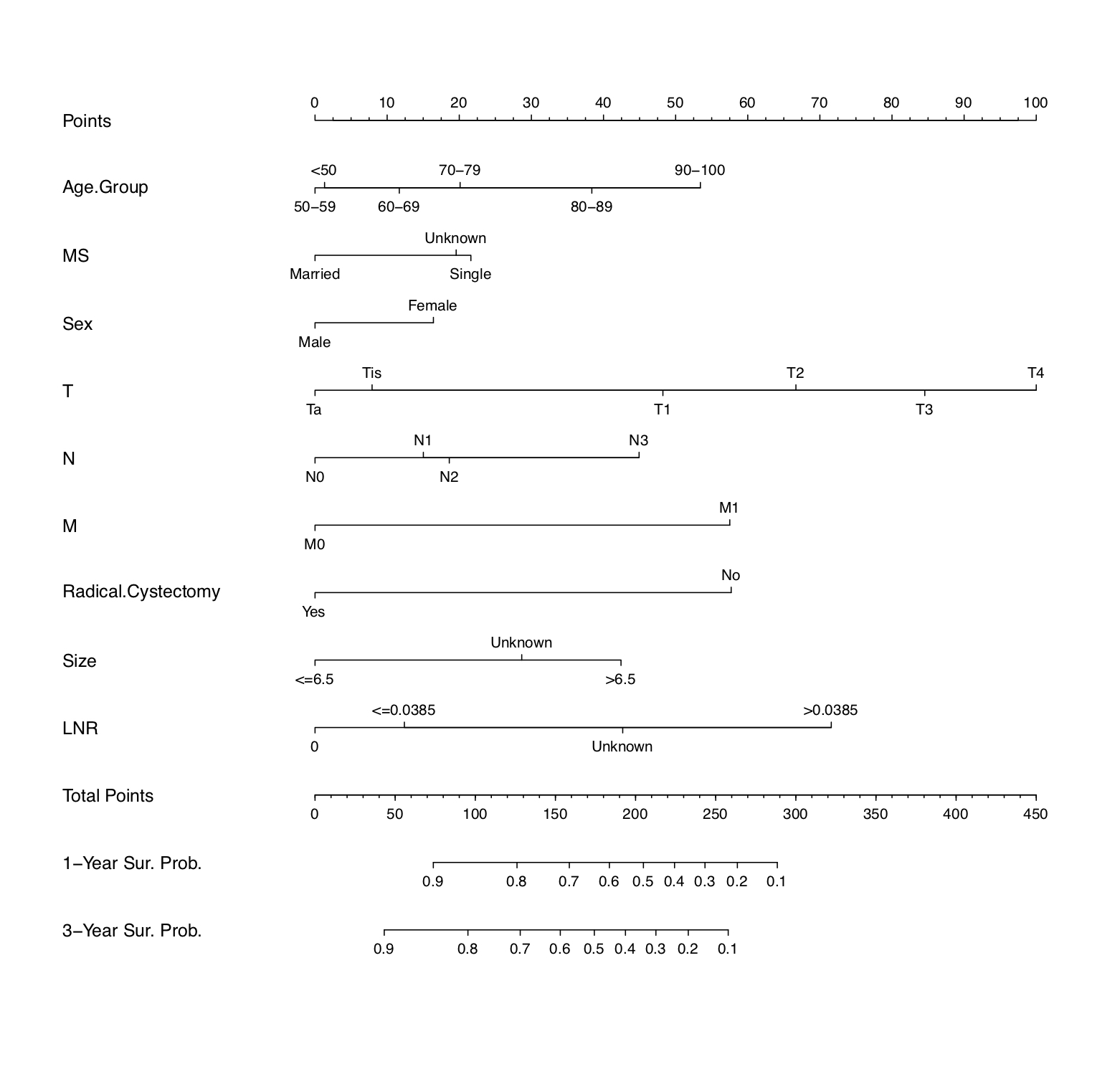

Supplement: Supplementary file 2 — Additional file 2 : Figure S2. Nomogram for predicting 1- and 3-year CSS of SCCB. Instruction of the nomogram: firstly, make a vertical line from certain variable to points scale to assign the point of that characteristic; then, add up all of the points from each characteristic and locating it to the total points’ scale; finally, draw a vertical line from the total points to 1- and 3-year CSS to predict the probability of CSS at 1- and 3-year. Abbreviations: CSS, cancer specific survival; MS, marital status; LNR, lymph node ratio; Sur. Prob., survival probability; SCCB, squamous cell carcinoma of the urinary bladder. [file 12885_2019_6430_MOESM2_ESM.jpg]

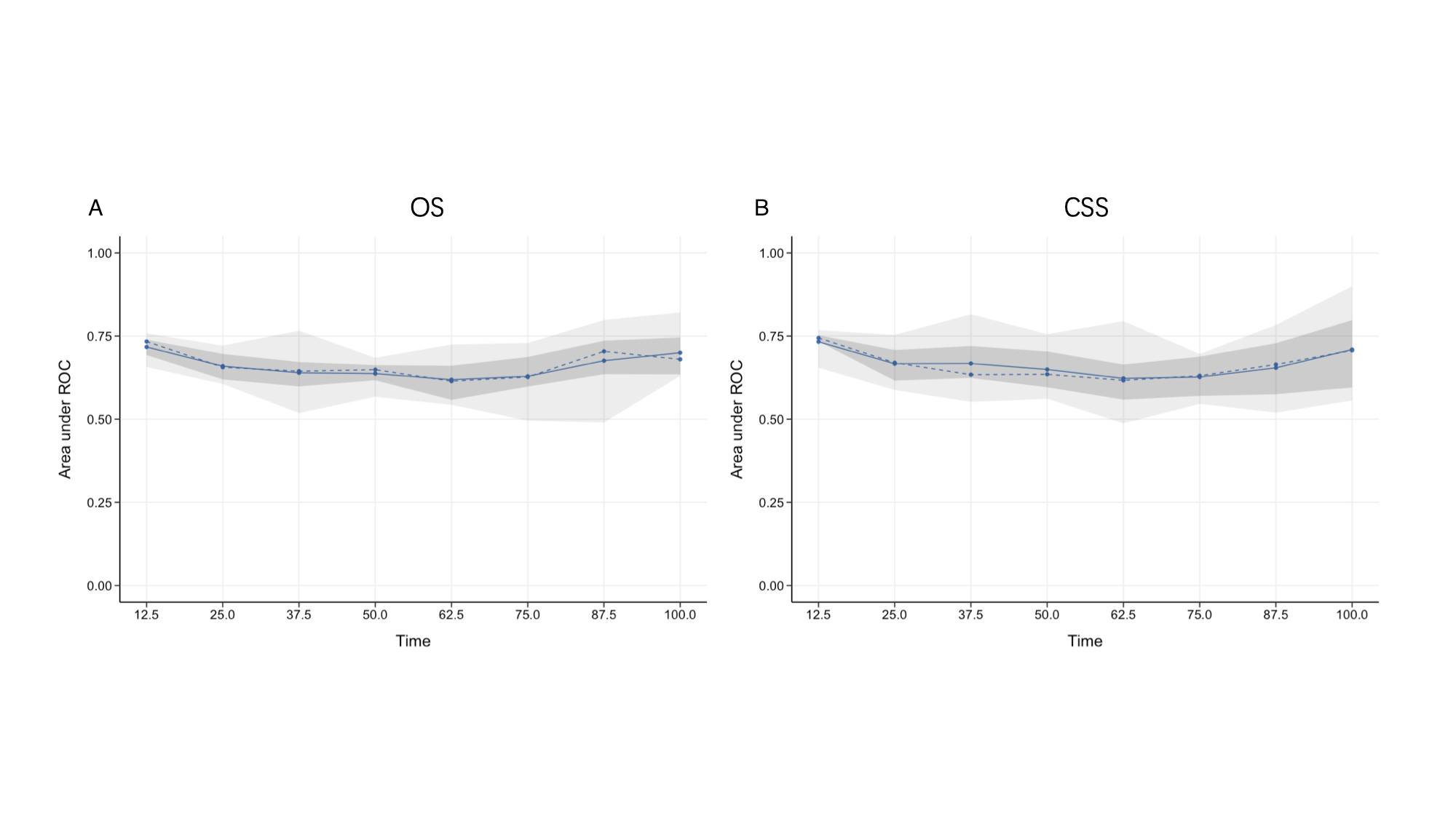

Supplement: Supplementary file 3 — Additional file 3 : Figure S3. Time-dependent AUC values for internal model validation. The 6-fold cross validation of (A) OS and (B) CSS. Abbreviations: AUC, area under the curve; OS, overall survival; CSS, cancer specific survival. [file 12885_2019_6430_MOESM3_ESM.jpg]
